# In Situ Coral Reef Oxygen Metabolism: An Eddy Correlation Study

**DOI:** 10.1371/journal.pone.0058581

**Published:** 2013-03-11

**Authors:** Matthew H. Long, Peter Berg, Dirk de Beer, Joseph C. Zieman

**Affiliations:** 1 Department of Environmental Sciences, University of Virginia, Charlottesville, Virginia, United States of America; 2 Max Plank Institute for Marine Microbiology, Bremen, Germany; Bangor University, United Kingdom

## Abstract

Quantitative studies of coral reefs are challenged by the three-dimensional hard structure of reefs and the high spatial variability and temporal dynamics of their metabolism. We used the non-invasive eddy correlation technique to examine respiration and photosynthesis rates, through O_2_ fluxes, from reef crests and reef slopes in the Florida Keys, USA. We assessed how the photosynthesis and respiration of different reef habitats is controlled by light and hydrodynamics. Numerous fluxes (over a 0.25 h period) were as high as 4500 mmol O_2_ m^−2^ d^−1^, which can only be explained by efficient light utilization by the phototrophic community and the complex canopy structure of the reef, having a many-fold larger surface area than its horizontal projection. Over diel cycles, the reef crest was net autotrophic, whereas on the reef slope oxygen production and respiration were balanced. The autotrophic nature of the shallow reef crests implies that the export of organics is an important source of primary production for the larger area. Net oxygen production on the reef crest was proportional to the light intensity, up to 1750 µmol photons m^−2^ s^−1^ and decreased thereafter as respiration was stimulated by high current velocities coincident with peak light levels. Nighttime respiration rates were also stimulated by the current velocity, through enhanced ventilation of the porous framework of the reef. Respiration rates were the highest directly after sunset, and then decreased during the night suggesting that highly labile photosynthates produced during the day fueled early-night respiration. The reef framework was also important to the acquisition of nutrients as the ambient nitrogen stock in the water had sufficient capacity to support these high production rates across the entire reef width. These direct measurements of complex reefs systems yielded high metabolic rates and dynamics that can only be determined through in situ, high temporal resolution measurements.

## Introduction

Coral reefs are among the most diverse habitats in the world, due to their high productivity and complex architecture that can shelter a wide diversity of organisms. Close to a billion people are to some extent dependent on reefs, and 275 million people live less than 30 km from the coast whose ‘*livelihoods are most likely to depend on reefs’* [1]. Therefore, it is important to understand the factors that govern reef productivity and ecosystem health, and such studies should preferably be done directly in the field under undisturbed conditions [2]. Many physiological studies have been done on reef species under defined conditions in the laboratory or in the field [3], [4]. However, the diverse species composition and the complex three-dimensional (3D) structures of natural reefs make it very difficult to extrapolate observations on single species under controlled conditions to a complete reef system.

The total surface area of reefs can be 15 times the planar reef area [5]. This surface area not only applies to the area available for photosynthesizers, but also to the reef framework which is the total of the pore spaces, cavities, and underlying sands that are important to the capture and transformation of nutrients in reef systems [6], [7], [8]. This high surface area of reefs, when examined in situ on an ecosystem scale, has the potential to produce much larger exchanges of solutes and organics than studies that give exchange rates that are normalized to organism surface area.

Reef metabolic rates are highly affected by the variable hydrodynamics and the numerous micro-habitats created by the reef's 3D structure. This makes it very difficult to extrapolate observations from a small area or a single species to a complete reef system. The 3D structure also creates micro-habitats that receive different amounts of light throughout the day [9] and different hydrodynamics that change with the current and wave direction. Numerous studies have shown the importance of hydrodynamics in stimulating photosynthesis through the enhanced O_2_ efflux from photosynthesizers and in increasing respiration due to the flushing of permeable sediments [10], [11], [12], [13]. For these reasons the need to sample is situ is highly important as it is the most accurate way to determine the realistic, integrated effect of these processes [2], [14].

Many of the in situ analyses of coral reefs have been done on shallow, low energy reefs with a unidirectional current, as most of the evaluations were done by flow respirometry techniques that require these conditions [2], [15]. While flow respirometry techniques are done in situ, they can only be applied in shallow water, are labor intensive, often take weeks to develop a single photosynthesis to irradiance (P-I) curve, and must account for the exchange of O_2_ across the water-air interface which is difficult to define accurately [2], [16]. For these reasons very little data exists for complete reef ecosystems. This lack of complete reef ecosystem analysis is further confounded by the variety of different reef organisms, environments, and surface areas that produce a wide range of gross primary production values from 30 to 1369 mol C m^−2^ y^−1^ [15]. With this large range in production, further research is needed to determine how the production varies under in situ conditions over different reef types.

In this study we used the eddy correlation (EC) technique [17] to examine the in situ dynamics of O_2_ production and consumption for the entire reef environment representing corals, algae, and other benthic organisms. The EC technique has proven very useful in a variety of environments including: muddy deep-sea sediments [18], permeable sediments [19], temperate seagrass beds [14], hard-bottom substrates [20], and Arctic sea-ice production [21], and has revealed in situ rates, dynamics, and interactions which cannot be observed by other methodologies.

The goals of this research were (1) to quantify O_2_ exchange in different reef habitats, including highly productive reef crests and less productive reef slopes and (2) to examine how production and respiration are affected by light, hydrodynamics, and community structure. To answer these questions we applied EC to the high surface area, rough hydrodynamic and heterogeneous reef environments. We finally discuss the suitability of EC for coral reef studies and show that examining O_2_ exchange rates at a high temporal resolutions on an ecosystem-scale is important to revealing reef metabolism dynamics.

## Materials and Methods

### Study Site

**Figure 1:**
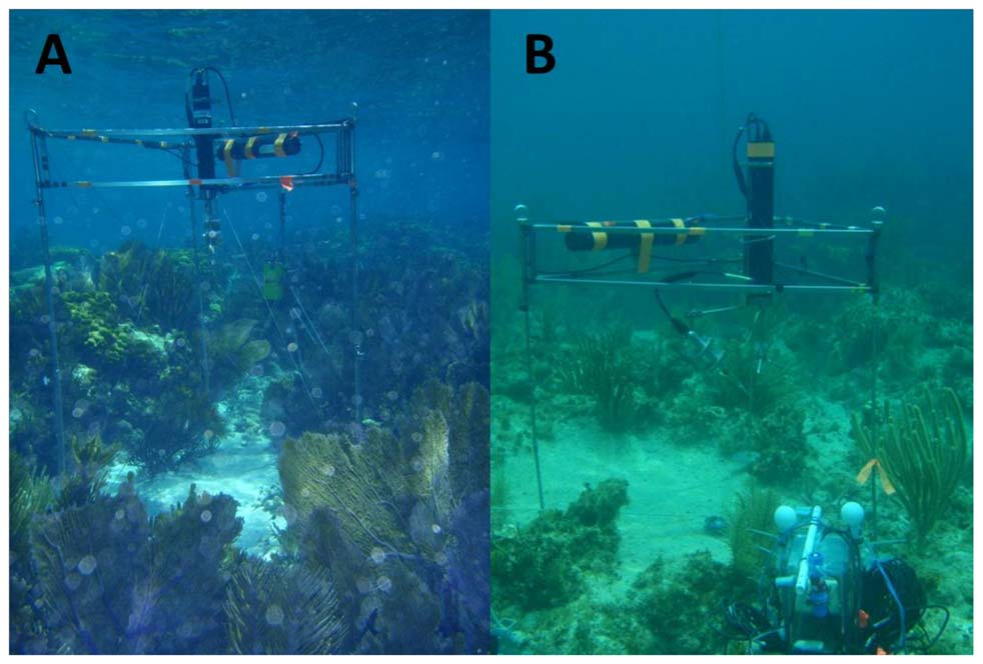
Eddy correlation instrument deployed on a reef crest (A) and reef slope (B) site. Figure 1A shows a reef crest site with a dense coverage of organisms over the reef structure and the dominance of gorgonians. Figure 1B shows a reef slope site and the dominance of rubble and sand, with a reduced coverage of organisms compared to the reef crest.

The research was done in the Florida Keys National Marine Sanctuary 7 km offshore within Grecian Rocks Sanctuary Protection Area (N 25°06′39′′, W 80°18′16′′) at the southern tip of Florida, USA. Research was conducted in June through August of 2009 and 2010. Two reef crest (RC) sites were located on the 2.0 m average depth reef crest and two reef slope (RS) sites were located 125 m away off the reef crest at an average depth of 4.5 m (Figure 1). The benthic communities of each site were characterized by transect analysis radiating away from each site in in 40 degree increments. Pictures were taken of 1 m^2^ quadrats by SCUBA diving at 0, 2, 5, 10, and 15 m, with the coverage of benthic communities determined by Coral Point Count with Excel extensions software [22].

### Eddy Correlation Measurements

The EC instrument consists of an Acoustic Doppler Velocimeter (ADV, Nortek AS, Norway), which measures the three dimensional velocity field and records the O_2_ concentration with a high-resolution custom-made picoamp amplifier (Max Planck Institute for Marine Microbiology, Germany). Fast responding (<0.3 sec) Clark-type O_2_ microsensors [23] that were designed to minimize stirring sensitivity (<2%) were attached to the amplifier and positioned at the edge of the ADV's 1 cm^3^ measuring volume [14], [17]. The velocity field and the O_2_ concentration were measured at 64 Hz over 0.25 h long measuring periods. Each deployment was >24 h to capture diel fluctuations. All instrumentation was self-contained and attached to a triangular frame that was designed to minimize hydrodynamic interferences. At all sites the instrument was located in a small sand patch with the measuring volume of the instrument located about 0.8 m above the bottom on the RC and 0.6 m above the bottom on the RS. A number of supporting instruments were also deployed to examine the drivers and controls of the 0.25 h averaged O_2_ fluxes. From the ADV measurements the current direction, mean velocities, and significant wave height were determined. Current direction was determined from the velocity field and a known instrument heading. Significant wave height was determined from the pressure data using QuickWave software (Nortek AS, Norway). Temperature was also recorded by the ADV. O_2_ optodes (Hach, USA) were positioned at the same height as the measuring volume and were used as a validation and calibration of the Clark-type microsensors. Photosynthetically active radiation (PAR) was evaluated at the same height as the instrument measuring volume using HOBO pendant light intensity loggers (Onset, USA) or LI-COR Spherical Quantum PAR Sensors (LI-193SA, LI-COR, USA) that were all site-specifically inter-calibrated as described by Long et al. (2012) [24]. The methods and analysis in Long et al. (2012) [24] show that HOBO light intensity loggers can be used to accurately mimic PAR sensors under the conditions at these sites. They also show that the calibrated PAR data from the HOBO loggers had an equivalent variability to that between two factory calibrated LI-COR PAR sensors.

### Data Analysis

The time-averaged flux across the reef-water interface was derived from high-resolution measurements of the vertical velocity and the O_2_ concentration as:



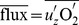
(1)


where the bars symbolize the averaging over time, and 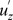 and 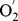 are the instantaneous fluctuating components of the vertical velocity and the O_2_ concentration, respectively [17]. The fluctuating components were determined by Reynolds Decomposition with the means determined by linear detrending over each 0.25 h measuring period [17]. The velocity and O_2_ data were carefully examined for sensor malfunctions due to particles or organisms interfering with the sensors which were evident by signal jumps, anomalous noise, variation from the stable optode, and loss of sensor response. Daily averaged rates of gross primary production (GPP), respiration (R), and net ecosystem metabolism (NEM) were calculated from 24 h long data sets where NEM = GPP+R. The daily rates were weighted by the hours of light and dark and assumed that the respiration rates were the same during the day and night [14], [16], [25].

The 0.25 h fluxes were examined for corrleations with measured environmental parameters using linear regression analysis. The daytime fluxes were also correlated to light using standard P-I curves which account for respiration [26], [27]. Binning was used to examine the average variations in the flux due to measured environmental parameters through binning by hour, velocity, temperature, significant wave height, and current direction. The linear correlations to the binned data were weighted by the standard error (SE) of each binned value [28].

Subsets of EC data were also examined for variances due to the averaging method used to determine the means in the Reynolds Decomposition including: mean removal, linear detrending, and filtering [18], [29]. The averaging window used for filtering was 60 sec. Wave and sensor tilt biases [30] were also investigated over the same time periods by rotating the 3D velocity coordinates at −5°, 0°, and +5° increments around the X and Y axes to examine the potential for any substantial bias. Data sets consisted of a continuous time series of similar magnitude fluxes. Standard one-way ANOVAs at the *p* = 0.01 level were used to determine if differences existed between the different data treatments. Rotation of the velocity field, where the X velocity is aligned with the mean current direction, was not done as Reimers et al. (2012) [31] reports that the rotation of the coordinate system in waves can cause large underestimate in the flux.

## Results

### Reef Metabolism

The transect analysis revealed that the reef crest (RC) sites were dominated by gorgonians and soft corals (51%), algae (24%), and sand, pavement, and rubble (20%) where the percentages are the percent cover of the reef surface. The reef slope (RS) sites were dominated by sand, pavement, and rubble (47%), algae (41%), and gorgonians and soft corals (9%). Hard coral coverage averaged 3% for the RC sites and 1% for the RS sites. The RC had a well-developed hard reef structure that was ∼0.5 m high over the underlying permeable sand, while the RS had scattered coral heads and rubble with permeable sand separating them (Figure 1).

**Figure 2:**
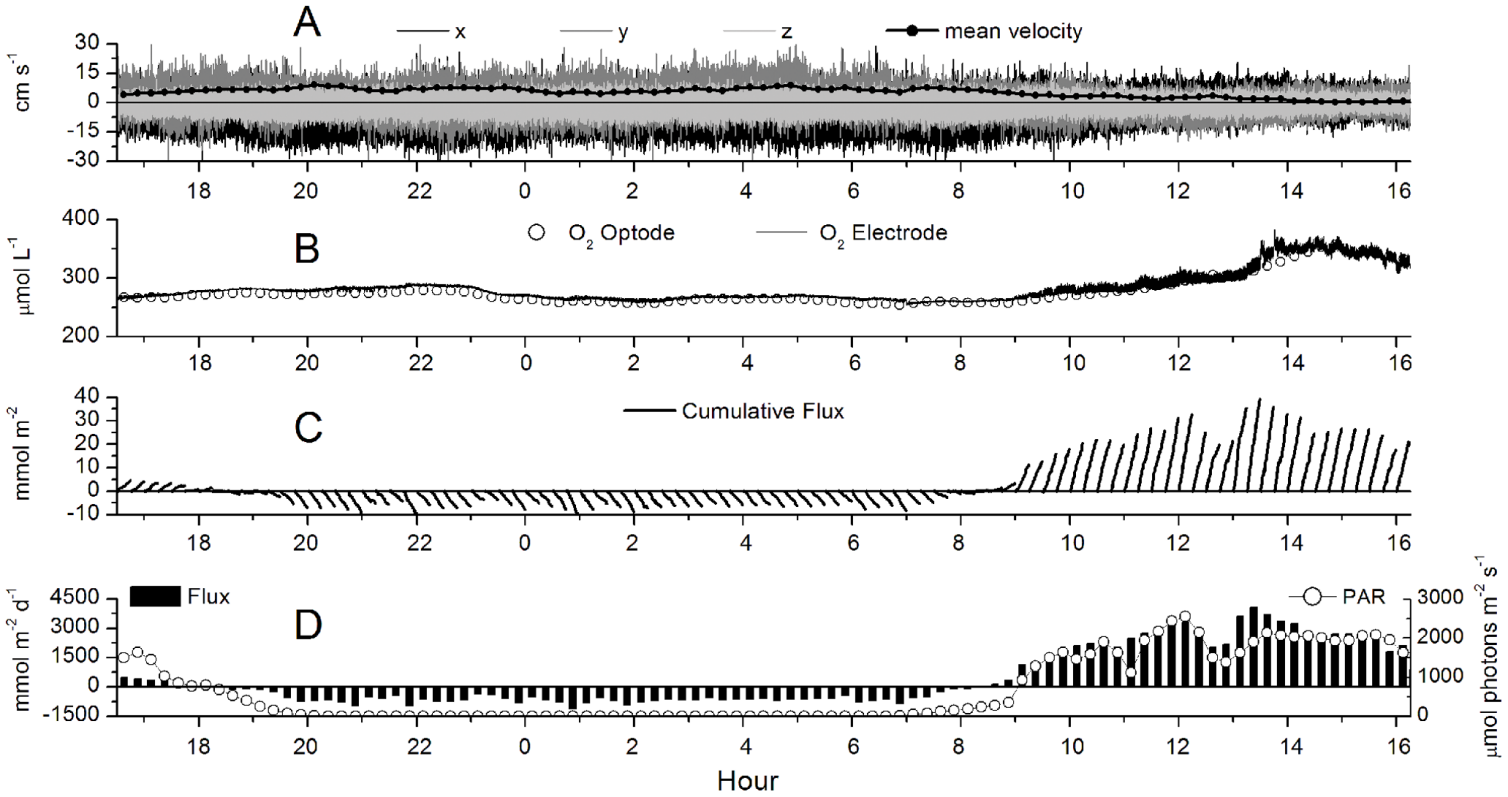
Example 24 hour EC data from the RC site. Figure A shows the 16 Hz *x*,*y*, and *z* components of the velocity and the mean total velocity. Figure B shows the 16 Hz O_2_ concentration and the O_2_ concentration from the optode. The cumulative O_2_ flux over each 0.25 h measuring period is shown in Figure C and the average flux and PAR measurements over each 0.25 h measuring period is shown in Figure D.

**Figure 3:**
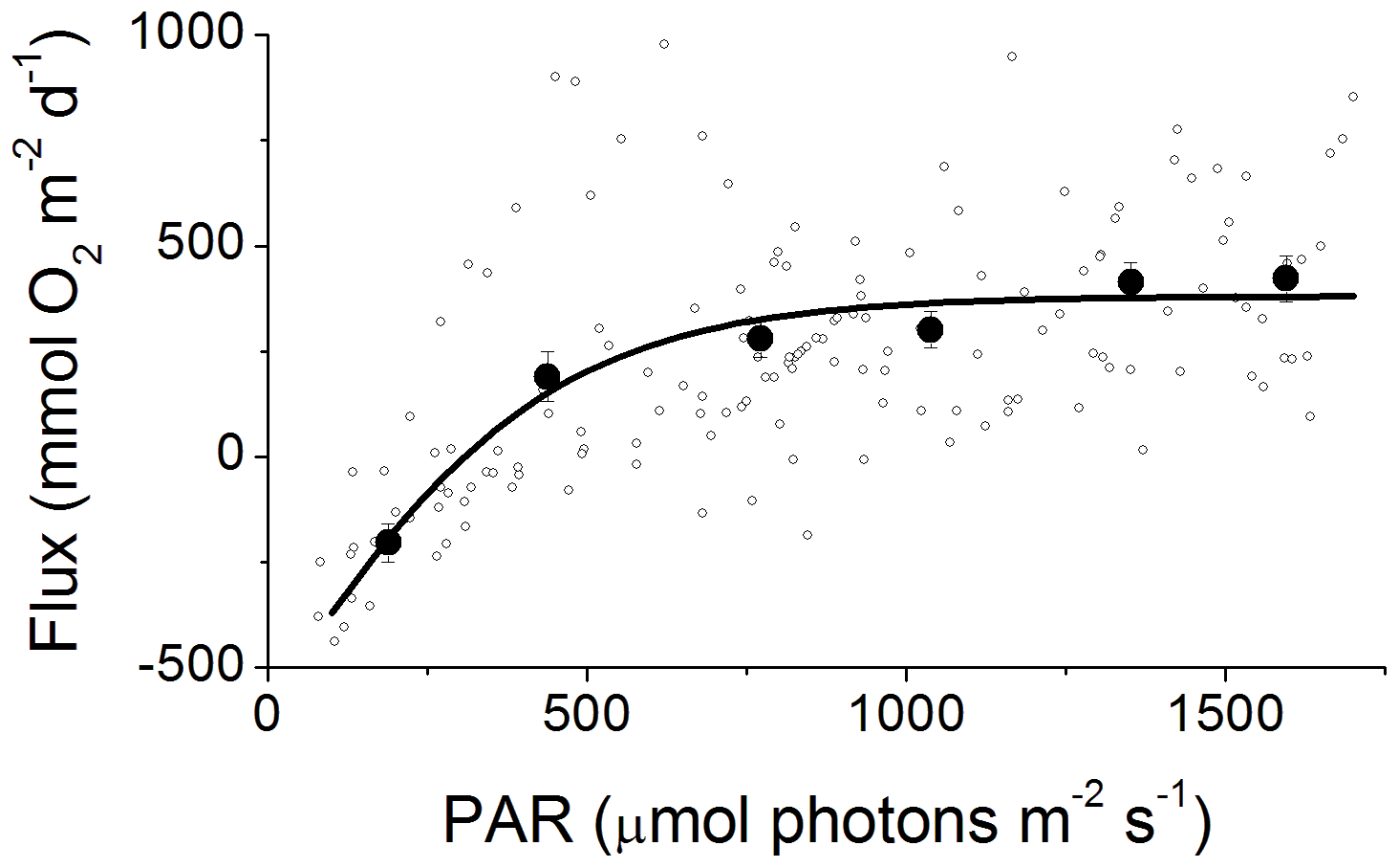
P-I curves for the RS site fit with a hyperbolic tangent funtion. The open circles represent the average flux over each 0.25 h measuring period during daylight hours. The solid circles represent binned averages over 300 PAR and the error bars are ±SE, with the line fit to binned data. From this curve a maximum production rate of 970 mmol O_2_ m^−2^ d^−1^, a light compensation point of 300 µmol photons m^−2^ s^−1^, and an intercept (approximating R) of −590 mmol O_2_ m^−2^ d^−1^, were found for the RS.

**Figure 4:**
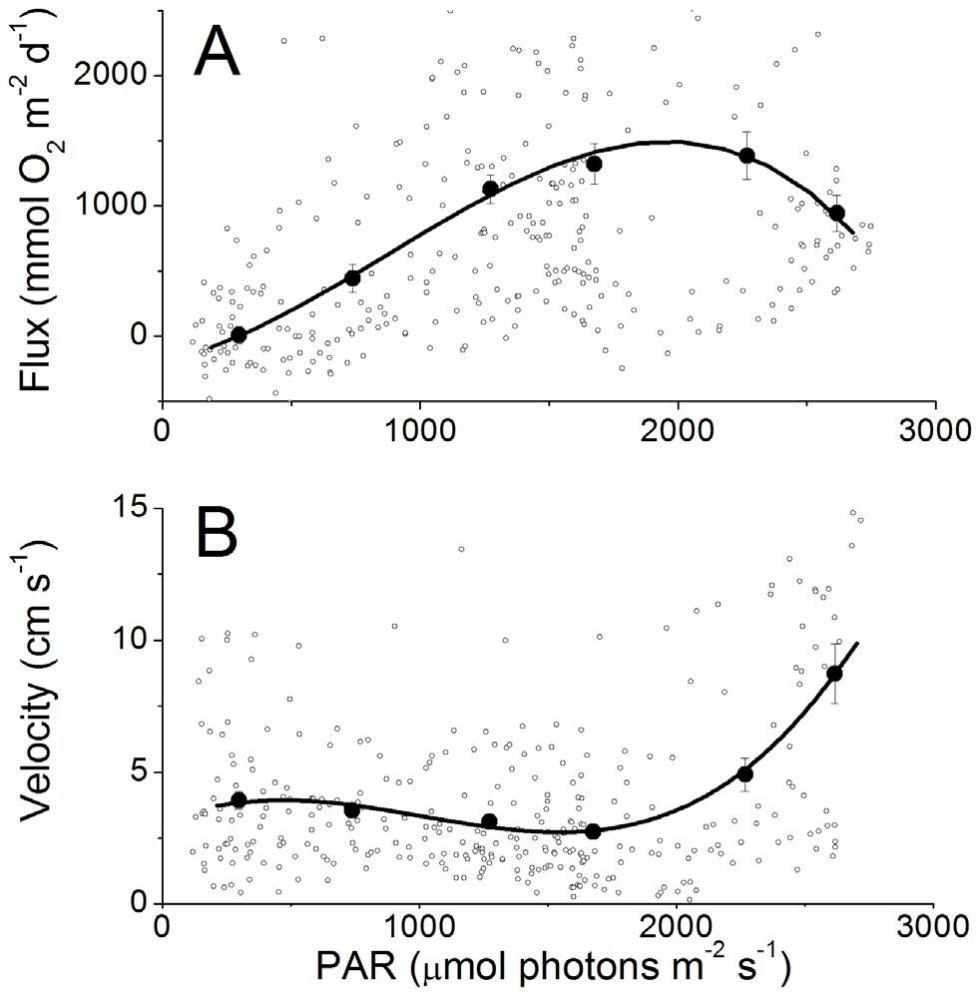
P-I curve for the RC site showing decreased production at high irradiences and high velocities. The open circles represent the average flux (Figure 3A) and the average velocities (Figure 3B) over each 0.25 h measuring period during daylight hours. The solid circles represent averages over 460 PAR and the error bars are SE with lines fit to binned data. The lines are a B-splines fit to the averaged data.

**Figure 5:**
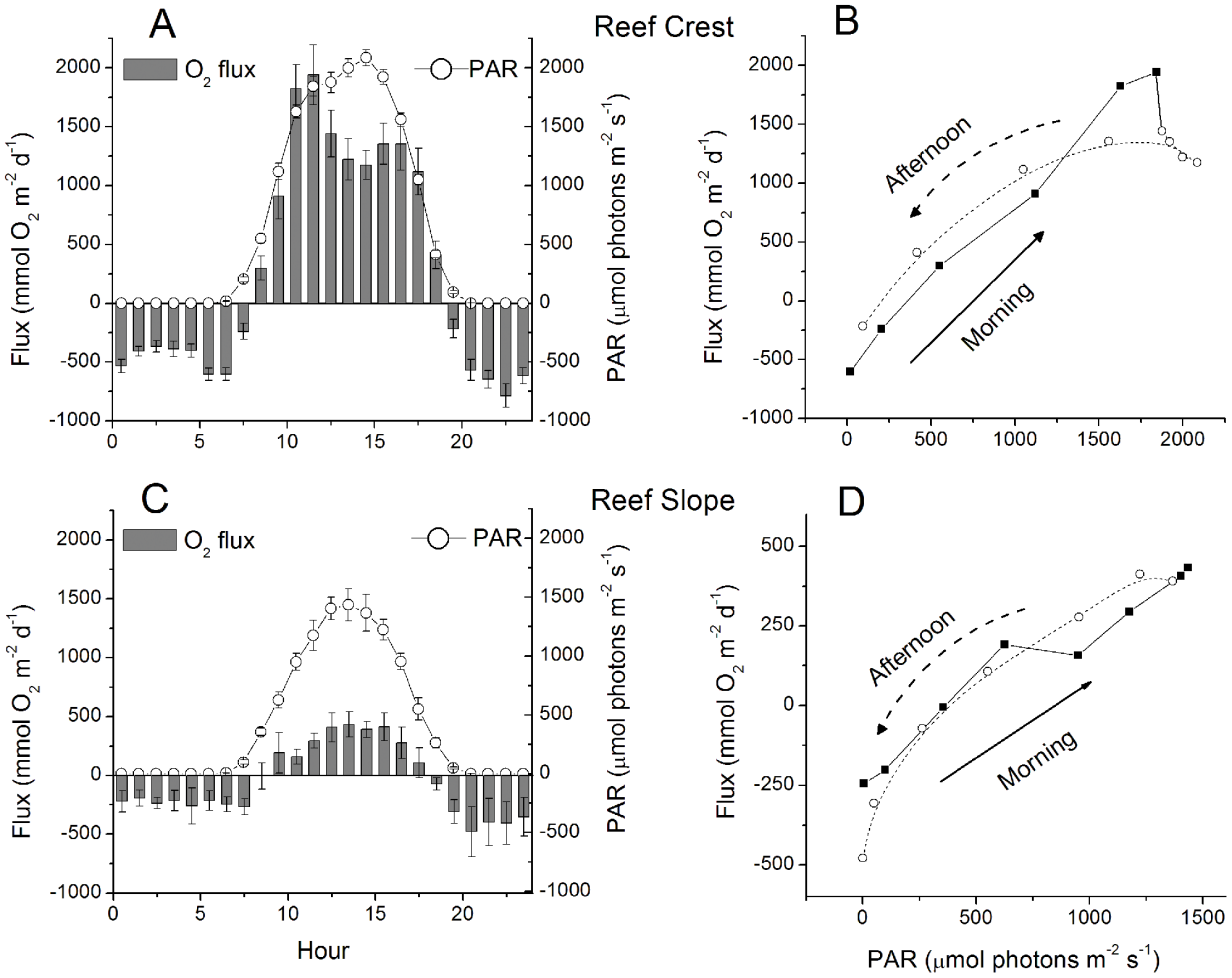
Hourly averages of O_2_ flux and PAR on the RC and the RS. The RC shows light inhibition at high irradiences which may be partially due to the stimulation of respiration through high velocities (Figure 4). Hourly averages of daytime production over 7 and 5 d for the RC (Figure 6B) and RS (Figure 6D), respectively. At both sites a linear increase in production is seen in the moring (solid circles) and an exponential decrease is seen in the afternoon (open circles). The morning and afternoon fluxes were not significantly different on the RC (*F_1_* = 0.0174, *p* = 0.895) or the RS (*F_1_* = 0.3277, *p* = 0.568). Error bars are omitted for clarity, note the difference in axis scales for Figure 6D.

An example of a 24 h EC data set for the RC is presented in Figure 2 and shows that the flux was highly dependent on the available light and reached fluxes of up to 4500 mmol O_2_ m^−2^ d^−1^ over a 0.25 h interval. When all the fluxes from multiple 24 h periods were binned and plotted versus light (Figure 3) a hyperbolic tangent fit modified for respiration [26] was found for the RS sites, producing a maximum average production rate of 385 mmol O_2_ m^−2^ d^−1^ (determined from the maximum measured PAR), a respiration rate of −590 mmol O_2_ m^−2^ d^−1^, and a light compensation point of 300 µmol photons m^−2^ s^−1^. However, on the RC sites we observed a decrease in the flux with the highest irradiances (Figure 4A) that were coincident with the highest velocities (Figure 4B). On the RC the flux increased linearly with light to about 1750 µmol photons m^−2^ s^−1^. The concurrent variation of the flow velocity and irradiance prohibited the analysis by standard P-I curves for the RC sites. The decreased oxygen production at the highest irradiances occurred in the afternoon (Figure 5A), when the flow velocities were also the highest. This was not found on the RS site (Figure 5C). When disregarding the afternoon depression on the RC, a linear increase in the flux with increasing irradiance (morning) was found at both sites and an exponential decrease in the flux with decreasing irradiance (afternoon) (Figure 5B and D, respectively). At both sites no significant effect of the temperature (varying from 28.4 to 31.8°C) was observed between the binned temperature and flux on the RC (R^2^ = 0.44, *p* = 0.155) or the RS (R^2^ = 0.35, *p* = 0.214).

**Figure 6:**
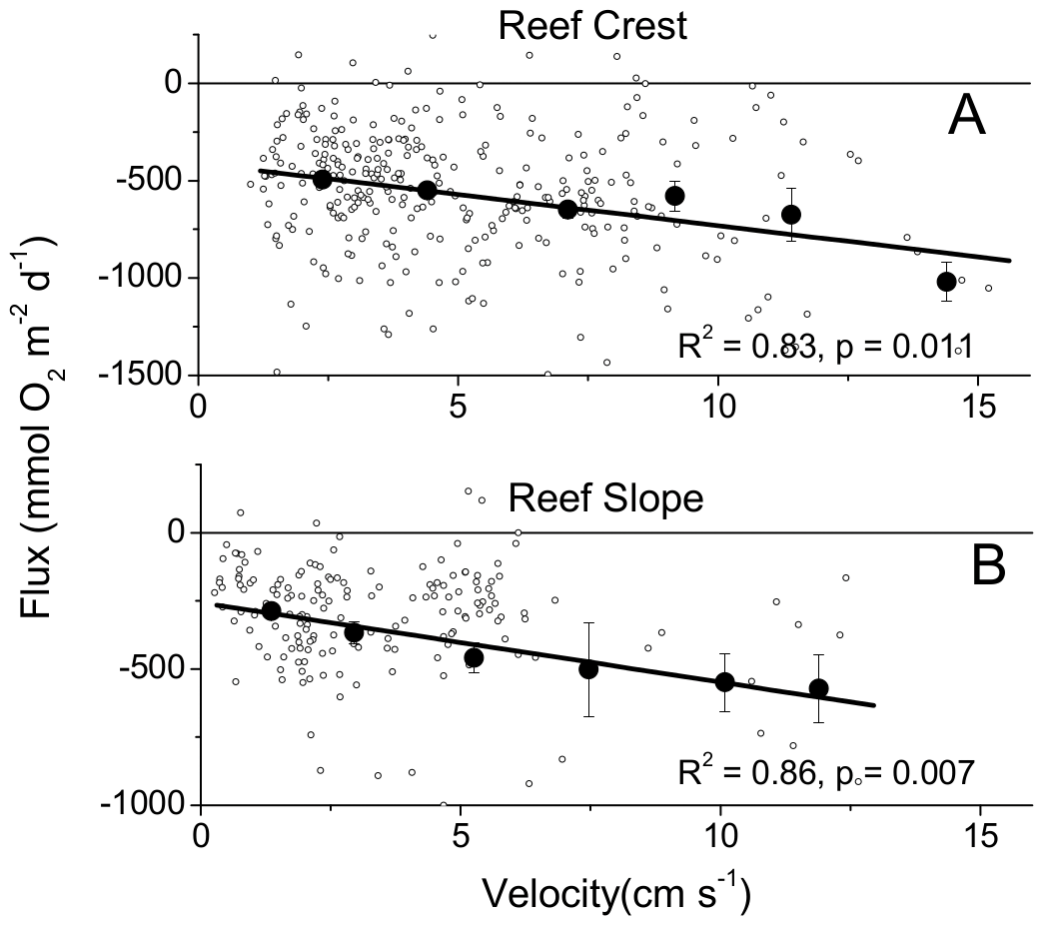
The increase in nighttime respiration correlated to increased velocity. Velocity was shown to increase the R rates at both the RC (A) and the RS (B). The open circles represent the average flux over each 0.25 h measuring period during the night. Lines are fit to binned data and error bars are SE.

**Figure 7:**
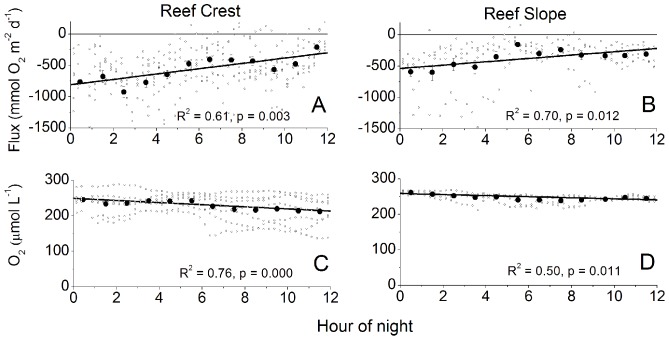
The large decrease in R but small decrease of O_2_ across the night. Figure A and B show the decrease in R across the night of approximately 60% at both sites. Figure C and D show a 5 to 15% decrease in the O_2_ concentration across the night which cannot explain the decrease in respiration. The solid circles represent averages over 1 h and the error bars are ±SE with the lines fit to binned data. Correlations applied to hourly binned data (± SE), with the small open circles representing the 0.25 h fluxes.

Nighttime respiration (R) rates were similarly increased by the velocity on both the RC and RS sites (Figure 6). Significant wave height did not produce changes in R rates on the RC (R^2^ = 0.285, p = 0.275) or the RS (R^2^ = 0.236, p = 0.329) when binned and correlated in a similar way. The R rates decreased gradually during the night on both sites (Figure 7A and B). This decrease in R rates across the night was coincident with decreases in the mean O_2_ concentration (Figure 7C and D). However, the decrease in the R rate was approximately 60% on both sites, while the mean O_2_ concentrations decreased by only 5–15%. The flow velocities and the wave heights did not change significantly across the night (data not shown). Thus, neither the small decrease in the O_2_ concentration, nor hydrodynamic effects were the cause for the decrease in respiratory activity.

**Figure 8:**
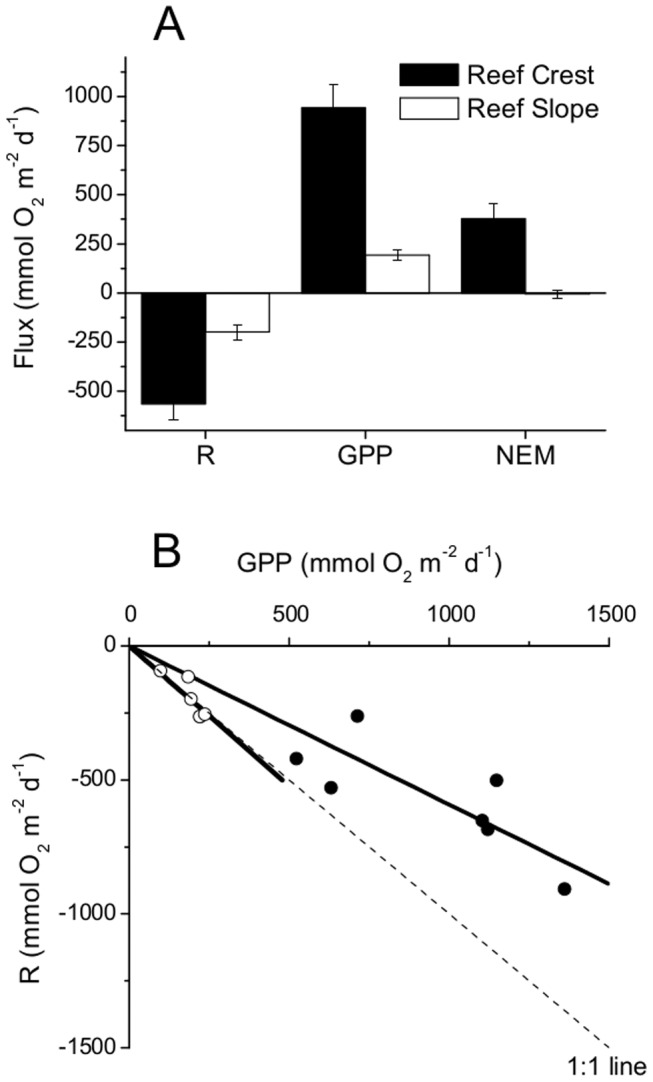
The average respiration, gross primary production, and net ecosystem metabolism at each site. The average respiration, gross primary production and net ecosystem metabolism rates across 24 h periods for the RC site (*n* = 7) and the RS site (*n* = 5) in Fig A. The *n* is the number of 24 h periods and the error bars are ±SE. The relationship between the net production and the nightly respiration over each 24 h period are shown in Figure B. The solid circles are the RC site and the open circles are the RS site.

Over a diel cycle, the RS had a net balance between respiration (R) and gross primary production (GPP), and a net ecosystem metabolism (NEM) of approximately 0, while the RC had a net positive NEM (Table 1, Figure 8A). All daily GPP and R correlations (each obtained from the same 24 h measurement, Figure 8B) show a consistent net balance between GPP and R for the RS and net positive rates for the RC. Thus, the RC was net autotrophic during the measurements, whereas on the RS the autotrophic and heterotrophic processes were equal.

**Table 1:** GPP, R, and NEM data for the RC and RS sites.

Site	*n*	GPP	R	NEM	*NEM ANOVA*	
		(mmol m^−2^ d^−1^)	(mmol m^−2^ d^−1^)	(mmol m^−2^ d^−1^)	*F_1_*	*P*
Reef Crest	7	944±120	−566±78	378±76	24.437	0.001
Reef Slope	5	193±25	−199±39	−6±21	0.073	0.794

ANOVAs (one-way, *p* = 0.01 level) tests on NEM difference from 0. The *n* is the number of 24 h measuring periods. Error values are standard error (±SE).

### Application of Eddy Correlation to Reefs

A total of 23 RC and 8 RS deployments were conducted, resulting in 7 complete RC 24 h data sets and 5 complete RS 24 h datasets. Due to the rough hydrodynamics and faunal activity at the RC sites microsensor breakage was common. Interference and breakage from large debris such as seagrass blades occurred periodically at both sites and microorganism biofouling on the glass O_2_ microsensors reduced the response times of electrodes after 48 h.

**Figure 9:**
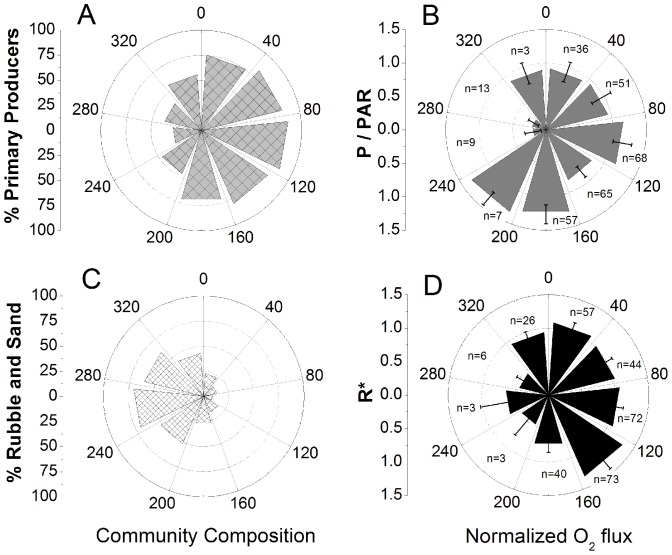
The flux as a function of direction and how it relates to the community composition. Panel A and C show the RC percent coverage of primary producers and percent rubble and sand in each direction, respectively, with the highest percentages of rubble and sand coincident with the lowest fluxes (as well as the lowest *n*). A directional plot showing the RC flux during the day divided by the PAR level is shown in panel B. Similarly, a directional plot of the RC flux during the night is shown in panel D, where R* is the instantaneous flux divided by the average flux. The *n* is the number of 0.25 h measuring periods in each direction and error bars are ±SE.

The footprint of the EC technique changed with the current direction and therefore the flux was examined as a function of direction on the RC (Figure 9). Using the transect analysis; polar plots were constructed showing the percentages of primary producers by direction (Figure 9A) and the percentage of rubble and sand by direction for the RC (Figure 9C). The daytime 0.25 h fluxes were divided by the irradiance, to account for the covariance of irradiance and the current direction (i.e. Figure 4), which enabled the examination of flux variations due to the changing footprint with current direction (Figure 9B). The nighttime 0.25 h fluxes were divided by the average R rate and plotted as a function of current direction (Figure 9D). For the RC site the lowest GPP and R rates were found from directions where the footprint was comprised of >40% rubble and sand, however approximately only 10% of the fluxes came from these directions. Thus the reefs were much more active than the surrounding areas. The RS footprints were more consistent with the percentage of primary producers being between 43 and 65% by direction and the percentage of rubble and sand being between 33 and 56% by direction and therefore no correlations with direction were observed or presented.

Variations in the EC data due to how the mean (used in the Reynolds Decomposition) was determined [18] were investigated by examining continuous data sets that had similar fluxes, velocities, and wave heights (Table 2). Similarly, an artificially imposed sensor tilt was used to examine whether the sensors were oriented normally to the flow field or if wave bias was present, as this tilt can cause a variation in the vertical velocities used to calculate the flux [30], [32]. None of the EC data was significantly different regardless of the mean determining method used or the artificial rotation of the coordinate system (Table 2). Therefore, the fluxes were not sensitive to wave or tilt biases, or the definition of the mean.

**Table 2:** ANOVA tests of EC data variation due to sensor tilt or mean determining method.

O_2_ Flux	*n*	Wave Height	Mean Flow	*Rotation ANOVA*		*Mean method ANOVA*	
(mmol m^−2^ d^−1^)		(cm)	(cm s^−1^)	*F_4_*	*p*	*F_2_*	*P*
3195±236	14	12.1±0.1	1.9±0.2	1.103	0.363	0.17	0.844
2471±246	16	ND	1.3±0.1	0.364	0.834	0.153	0.859
1810±232	20	11.8±0.2	3.7±0.3	1.206	0.314	0.099	0.906
896±138	24	15.7±0.3	1.4±0.1	1.304	0.273	0.028	0.972
880±105	13	11±0.4	0.5±0.1	0.616	0.653	2.106	0.141
−475±80	13	11.7±0.3	1.2±0.1	0.934	0.451	0.309	0.737
−544±40	13	17.5±0.4	0.4±0.1	2.427	0.060	3.410	0.044
−726±116	11	13.6±0.6	7.7±0.3	1.725	0.160	0.574	0.569
−730±43	14	16.5±0.3	5.8±0.2	2.113	0.089	3.470	0.041
−1280±113	22	ND	4.1±0.2	0.587	0.673	0.160	0.853

ANOVAs (one-way, *p* = 0.01 level) tests on subsets of EC data with similar O_2_ fluxes, significant wave heights and mean velocities. Rotation ANOVA is for rotations of +5° in X and Y, −5° in X and Y, and no rotation. Mean method ANOVA is for means determined by mean removal, linear detrending, and filtering. The *n* is the number of 0.25 h measuring periods. Error values are standard error (±SE). ND indicates no data.

## Discussion

### Reef Primary Production

The measured areal O_2_ fluxes were up to 4500 mmol m^−2^ d^−1^ (Figure 2), which is much higher than reported for reef species as measured under laboratory conditions (ca 900 mmol m^−2^ d^−1^, [9]). Also different from common incubation experiments is that the P-I curve did not show saturation under light intensities of over 1750 µmol photons m^−2^ s^−1^ (Figure 4A), whereas even light adapted corals are typically saturated at 200–300 µmol photons m^−2^ s^−1^ [33]. The much higher areal productivity and the absence of light saturation can be explained by the complex 3D structure of coral reefs. Coral reefs can have a surface area to reef planar area ratio of up to 15 for well-developed reefs [5]; therefore, small scale measurements cannot be simply extrapolated to the entire reef ecosystem. In a coral reef a range of light microenvironments are present and vary by their position in the canopy structure [6], [9]. Therefore, light coming into the canopy is spread out over a much larger surface area than that of the reef planar surface and is used by a range of ambient light-adapted organisms. As the clear, tropical water absorbs hardly any light, the light that becomes increasingly scattered deeper in the canopy will be almost entirely attenuated by photosynthetically active surfaces, by low-light adapted organisms deeper in the reef. Thus direct and diffuse light can be efficiently harvested by phototrophs on reefs without loss by absorption by minerals or sediments as in microbial mats or microphytobenthos [34].

**Figure 10:**
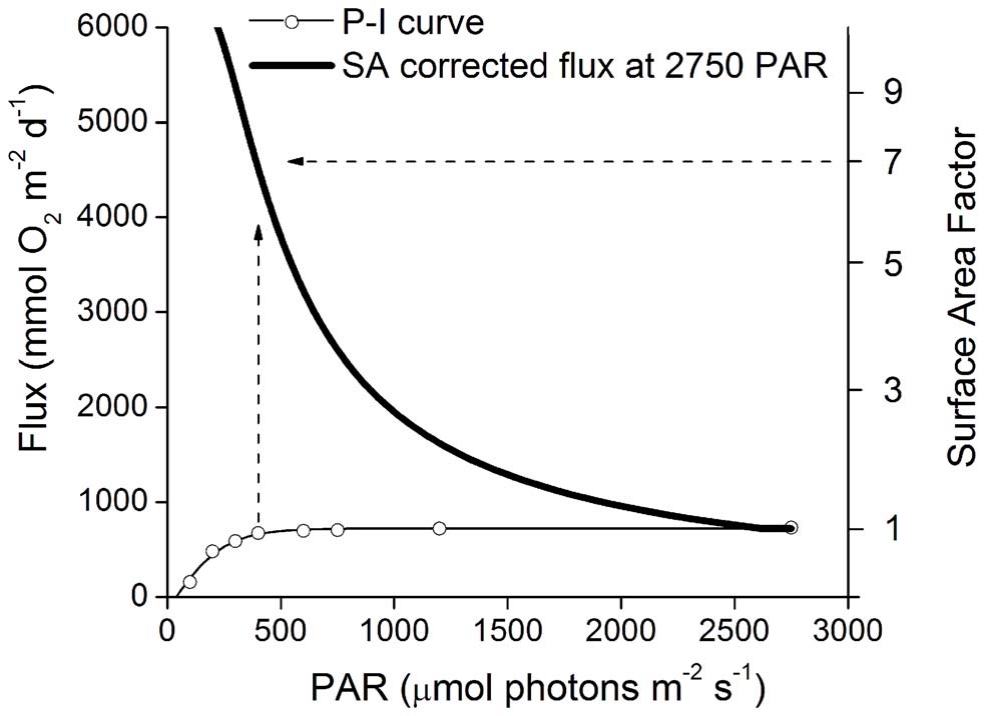
Standard P-I curve corrected for reef surface area during peak irradience. The dashed arrows show the construction of the surface area corrected flux during PAR conditions of 2750 µmol photons m^−2^ s^−1^. For example at a surface area factor of 7, 2750 PAR is spread out over 7× the area (i.e. 393 µmol photons m^−2^ s^−1^) and then the flux at this PAR level (? 650 mmol O_2_ m^−2^ d^−1^) is corrected for by multiplying by the surface area resulting in a flux of up to 4550 mmol O_2_ m^−2^ d^−1^. P-I curve adapted from Anthony and Hoegh-Guldberg (2003)[9].

Mesocosm and incubation methods commonly measure O_2_ exchange rates that are normalized to the total organism surface area. However, when O_2_ exchange rates are extrapolated to a larger area that accounts for the surface area to reef planar area ratio, these values can be much greater. For example, a hyperbolic P-I curve was adapted from Anthony and Hoegh-Guldberg (2003) [9] to show how the surface area of reefs can produce a much larger flux than experiments conducted ex situ and normalized to organism surface area (Figure 10). The P-I curve has a maximum production rate of 860 mmol O_2_ m^−2^ d^−1^ and a light saturation constant of 240 µmol m^−2^ d^−1^. However, when surface area is considered the light is spread out over a much larger area on a complex reef structure (per m^−2^ of planar reef area), and much higher rates of production are possible compared to that of a flat surface. For example, based on this first-order calculation, a reef with a surface area of 7 times that of the m^−2^ reef planar area may produce an average flux of 4550 mmol O_2_ m^−2^ d^−1^ at the peak light levels of 2750 µmol photons m^−2^ s^−1^ measured in this study (dashed arrows, Figure 10). Moreover, the reef community as a whole will not be easily saturated by light. While individual colonies at the top of the canopy may respond typically to a saturation P-I curves under increasing light intensities, deeper areas inhabited by low-light adapted phototrophs will be illuminated, and will efficiently use the increased number of photons for photosynthesis. Therefore, the response of a whole reef to increasing light will result in increased oxygen production, even when the species high in the canopy are saturated with light.

The shallow, well developed RC produced large fluxes during high irradiances (up to 2750 µmol photons m^−2^ s^−1^), with the daytime fluxes tightly coupled to the irradiance (Fig 2, 3A). While periods of high magnitude fluxes were observed daily, the maximum daily GPP rate was<1400 mmol O_2_ m^−2^ d^−1^ (Figure 8B). This daily integrated rate is similar to other studies on shallow reefs that have reported daily integrated GPP rates of up to 1730 mmol O_2_ m^−2^ d^−1^ [35], 2305 mmol O_2_ m^−2^ d^−1^ [36], 3600 mmol O_2_ m^−2^ d^−1^ [37] as well as yearly GPP rates up to 3750 mmol O_2_ m^−2^ d^−1^ [15] (assuming a 1∶1 ratio of C:O_2_ for comparison purposes). These studies were conducted using flow respirometry techniques which, like the EC technique, integrate over a large reef area and incorporate the complex surface area of coral reefs but do not have the spatial or temporal resolution of EC. The high-resolution and high-magnitude fluxes reported here can only be resolved by using the EC technique.

A substantial nutrient supply is required to support these large production values in the oligotrophic subtropical waters. A GPP rate of 1000 mmol C m^−2^ d^−1^ (assuming a O_2_:C ratio of 1∶1) and a conservative C:N ratio of 10∶1 for the reef benthos dominated by soft corals and macroalgae [38], [39] corresponds to a N requirement of ∼100 mmol N m^−2^ d^−1^. Using our average measured velocity of 0.04 m s^−1^, a 2 m deep and fully mixed water column, and a total N concentration measured over a nearby reef at the same time of year of 7 µmol L^−1^ [40], the rate of total N available per meter width of reef is 560 µmol total N s^−1^ m^−1^. While the inorganic N fraction represents only 2% of the total available N [40], the removal of particulates through grazing is thought to be the major mechanism by which external nutrients are transported into reefs [6], [41], [42]. This simple first-order calculation also assumes that no internal recycling of N occurs and that no excess N is provided via N fixation. The calculation reveals that 0.2% of the total N can support this level of production on a m^−2^ basis. This is equivalent to the removal of 36% of the total N across the entire reef width (∼175 m). Further, the high rates of plankton depletions reported for reefs of up to 90% [43], [44] suggests that a complete removal of N can support a reef up to about 480 m wide. A survey of similar shallow reefs in the area (estimated from Google Earth) is in line with this finding with all comparable reefs having a maximum width of <400 m.

### Drivers of Reef Production

The relationship between the irradiance and the daytime fluxes produced ecosystem-wide P-I curves with the RS displaying an expected production maxima at high irradiance levels (Figure 3). However, the RC produced a linear trend over the same irradiance levels observed on the RS (up to 1750 µmol photons m^−2^ s^−1^, Figure 4A). This is likely due to the extensive canopy structure on the RC that allows for more efficient light utilization whereas the RS with its reduced canopy could not use light as efficiently.

The reduced canopy structure on the RS may allow for photoinhibition which has been shown to reduce afternoon production rates as the photoreceptive capacities of phototrophs are reduced with increased light [45], [46], [47]. While photoinhibition may have been present on the RS, the reduced production at the highest irradiances on the RC was confounded by the coincident increase in afternoon flow velocities (Figure 4). The high amount of self-shading present in the RC canopy likely reduce photoinhibition as self-shading has been shown to increase ecosystem-wide production rates compared to individual species P-I relationships [48], [49]. A reduction in photoinhibition and increased production may also have occurred due to the flow-enhanced efflux of O_2_ from organisms [11], [12], [13], [50]. Therefore, the high flow conditions and shading effects of the RC canopy likely decreased photoinhibition. Thus, the enhancement of R by flow through the porous reef structure and underlying sand (Figure 6), combined with the daytime abundance of photosynthates, likely caused the daytime decrease in net O_2_ production during periods of high flow in the afternoon. Further, the fact that no increase in O_2_ production was found with increasing flow velocities suggests that the flow stimulation of R masked any concurrent increase in O_2_ production due to flow that may have occured. Compared to studies conducted on the surfaces of individual species, it is likely that the stimulation of R by flow seen here was much greater than the concurrent effects of flow stimulating photosynthesis when these processes were examined in situ on an ecosystem-scale.

While there were not significant differences between the morning and afternoon fluxes, a hysteresis was seen in the fluxes with morning fluxes increasing linearly with irradiance (Figure 5B and D). With the exception of the afternoon depression on the RC, the afternoon fluxes exhibited an exponential decrease in the production with decreasing irradiance. This suggests that photosynthetic organisms acclimate to high irradiance levels across the day but become less efficient at utilizing light as irradiance decreases. A similar result was also found by Levy et al. (2004) [46] where coral P-I curves had a much steeper initial slope in the afternoon than in the morning which is consistent with our exponential decrease with decreasing afternoon irradiance. It is also possible that enhanced rates of afternoon respiration [51] due to increases in available photosynthates [52] contribute to the exponential decrease in the net production rates when combined with decreasing irradiances.

### Reef Respiration Dynamics

Respiration is stimulated by flow due to the flushing of permeable sediments [10], [53], [54] and was evident on both the RC and RS at night (Figure 6). The larger R rates at the RC, compared to the RS, were likely due to the increased number and biomass of organisms present given the higher surface area on the exposed reef and porous reef framework. This hidden biomass in reef pore-spaces has high R rates and is dependent on the transport of dissolved gasses, nutrients, and organic matter into the cavities [6], [7], [8]. Therefore, these cavity-dwelling organisms that were more abundant on the RC were stimulated under high flow conditions evident in both the day and night (Figure 4 and 6).

Respiration rates also decreased across the night at both sites (Figure 7) which can be due to the decreased availability of O_2_ and photosynthates as they are consumed across the night or due to diel changes in the activity of reef heterotrophs [55]. The O_2_ concentrations on the RC and RS decreased by only 5–15% across the night while the R rate decrease by 60%. This decrease in R across the night is similar to that found by Falter et al. (2011) [55] of 77% on a reef flat, which was attributed to the build-up of photosynthates during the day. The abundance and activity of reef heterotrophs is greatest at dusk and dawn [56] and cannot explain the linear decrease in R across the night, nor the lower R rates at dawn. Therefore, the majority of the decrease in R observed across the night here was likely due to the reduced availability of photosynthates across the night, as no correlations with other physical parameters that are known to increase R were found (e.g. temperature, velocity, significant wave height).

The higher rates of R immediately after dark (Figure 5A, 5C, 7) also suggested that the daytime R rates would be greater due to readily available photosynthates [57]. This is supported by studies that have been able to separate daytime R from production and concluded that daytime R may be underestimated by up to 100% when assuming that R is equivalent during the day and night [57], [58], [59]. However, the daily rates of R determined here from nighttime values only represent conservative estimates, and may lead to an equivalent underestimate in daily GPP rates as they are calculated using this constant R rate. However, these conservative estimates do not affect the NEM as the NEM depends only on the summation of the fluxes over a 24 h period. Therefore, the daily NEM rates and the 0.25 h O_2_ fluxes represent the most accurate in situ analysis that can currently be conducted on reef ecosystems.

The RS had reduced GPP and R rates compared to those of the RC (Figure 5, Figure 8A) due to the greater depth (i.e. reduced light levels) and the reduced surface area, reef pores spaces, and primary producers present on the RS. On average the R and GPP on the RS were in balance while the RC was net autotrophic, where the latter is supported by numerous studies that show reef crests are typically autotrophic [15], [55], [60]. The balanced nature of the RS environment is also likely affected by the import of organic material with the RS acting as a filter of the exported organic material from the highly productive RC. The balanced conditions for the RS and autotrophic conditions for the RC are also strongly supported by the consistent daily correlations between R and GPP (Figure 8B) and suggests that increased production results in increased respiration [55], [61]. The consistent ∼1∶1 relationship (GPP:R) for the RS and the consistently >1∶1 ratio (GPP:R) for the RC illustrate the differences between a reef crest and that more characteristic of a less-developed reef slope habitat. This distinction is important as most measurements of total ecosystem coral reef metabolism have been done on reef crests [15]. However, reef slope environments can represent up to 85% of the total reef area [62] and therefore the examination of primarily reef crest environments in the past has misrepresented the primary production of reef communities [15].

### Reef Eddy Correlation

This research shows the EC technique as an excellent approach for the analysis of reef systems, as it can be deployed at any depth, is totally self-contained, and can be deployed over any bottom structure. The EC technique measures over a large reef area, or footprint, [63] and therefore integrates over the complex 3D reef structure. Therefore, O_2_ flux rates by EC are reported per m^2^ of planar surface, but incorporate the total 3D surface and structure of complex environments such as coral reefs. Through these measurements, high-temporal resolution O_2_ fluxes can be correlated to changes in environmental conditions while incorporating all of the organisms within and on the reef structure.

Sampling at this community level is also important due to the difficulty of scaling up measurements done on individuals to the community level, as they do not account for varying community compositions, self-shading, densities of individuals, 3D structures, or are done on only part of an individual [48], [49]. Therefore, the scaling up of rates that are normalized to organism surface, do not include all organisms, or are done in a single micro-environment likely cause underestimates in the metabolic activity of complex coral reefs. Thus, the in situ analysis of a wide spatial area that incorporates all of the variation in benthic communities and surface area is required to determine the true O_2_ flux rates for a complete reef system. For these reasons, these first eddy correlation data from reefs likely represents the most accurate representation of the O_2_ exchange rates of complete reef ecosystems.

The heterogeneous nature of reefs, combined with a changing EC footprint due to variations in the mean current direction, requires an analysis of the flux as a function of direction. The EC footprint is typically large, but is also narrow with a width on the order of a few meters [63]. Therefore, even small changes in the current direction can yield an entirely different footprint. The heterogeneous RC community structure caused variations in the O_2_ flux with changes in current direction (Figure 9). For example, when the footprint was oriented toward areas with a low percentage of primary producers, a reduced O_2_ production was measured (Figure 9A and B). Likewise, when the footprint was oriented toward areas dominated by sand and rubble, the R rates decreased (Figure 9C and D). These results illustrate that community heterogeneity must be considered when sampling over heterogeneous environments with EC, as well as other methods dependent upon current direction, such as flow respirometry techniques.

The stable and linear nature of the cumulative fluxes (the summation of the instantaneous fluxes) (Figure 2C) indicated a constant, strong flux signal from the reef community, representing quasi-steady state conditions over each 0.25 h measuring period [18]. These fluxes are transported across the reef-water interface by turbulent fluctuations [17] and when turbulent fluctuations in the velocity decrease, a corresponding increase in the fluctuations of O_2_ must occur to maintain the magnitude of the O_2_ flux. This can be seen in Figure 2A and 2B (hours 9–12), where a decrease in current velocity resulted in reduced turbulent mixing (Figure 2B). Specifically, at hour 9–10 the variance is 6.5 cm^2^ s^−2^ and 21.7 µmol^2^ L^−2^ for the vertical velocity and O_2_ concentration, respectively, while at hour 11–12 the variance is 5.3 cm^2^ s^−2^ and 34.5 µmol^2^ L^−2^, respectively.

If the velocity sensor is tilted relative to mean flow field of velocity, or if waves are present, a wave or tilt bias may occur [30], [32], [64]. These potential biases were examined by imposing ±5° rotations of the velocity field around the X and Y axes over time periods of different velocities and significant wave heights (Table 2). None of the rotations produced significant differences from the unrotated data and these results indicate that the presented EC measurements are not influenced by wave or tilt biases. Three different methods of mean determination (mean removal, linear detrending, and filtering; [18]) used in the Reynolds Decomposition of the velocity and O_2_ measurements were also examined and did not produce any significant differences in the flux (Table 2). This indicates that all three methods were suitable for calculating the flux under the conditions present on the reef.

### Conclusions

These results highlight the amount and quality of data that can be measured with EC while showing that it can be applied in complex and challenging environments. These data revealed high production rates on autotrophic reef crests due to high SA that created numerous microenvironments relative to light and flow. This high primary production and 3D structure of reefs also supports a large range and quantity of organisms, enabling reefs to be hotspots of biodiversity and a source of primary production to surrounding environments. These ecosystem-scale measurements also show how high production rates are possible, even in nutrient-replete environments, when the reef canopy and framework are considered. Furthermore, when working in complex environments, ecosystem-scale measures that include all variability in the system are needed to determine the net effects on production, respiration and ultimately carbon cycling.
